# Long-term climate establishes functional legacies by altering microbial traits

**DOI:** 10.1093/ismejo/wraf005

**Published:** 2025-01-13

**Authors:** Caitlin M Broderick, Gian Maria Niccolò Benucci, Luciana Ruggiero Bachega, Gabriel D Miller, Sarah E Evans, Christine V Hawkes

**Affiliations:** W.K. Kellogg Biological Station, Michigan State University, 3700 Gull Lake Drive, Hickory Corners, MI 49060, United States; Department of Integrative Biology, Michigan State University, East Lansing, MI 48824, United States; Department of Plant, Soil, and Microbial Sciences, Michigan State University, East Lansing, MI 48824, United States; Department of Plant and Microbial Biology, North Carolina State University, Raleigh, NC 27607, United States; Department of Integrative Biology, University of Texas at Austin, Austin, TX 78712, United States; W.K. Kellogg Biological Station, Michigan State University, 3700 Gull Lake Drive, Hickory Corners, MI 49060, United States; Department of Integrative Biology, Michigan State University, East Lansing, MI 48824, United States; Ecology, Evolution, and Behavior Program, Michigan State University, East Lansing, MI, United States; Department of Plant and Microbial Biology, North Carolina State University, Raleigh, NC 27607, United States; Department of Integrative Biology, University of Texas at Austin, Austin, TX 78712, United States

**Keywords:** microbial traits, climate legacy effects, functional potential, metagenomics, microbial ecology

## Abstract

Long-term climate history can influence rates of soil carbon cycling but the microbial traits underlying these legacy effects are not well understood. Legacies may result if historical climate differences alter the traits of soil microbial communities, particularly those associated with carbon cycling and stress tolerance. However, it is also possible that contemporary conditions can overcome the influence of historical climate, particularly under extreme conditions. Using shotgun metagenomics, we assessed the composition of soil microbial functional genes across a mean annual precipitation gradient that previously showed evidence of strong climate legacies in soil carbon flux and extracellular enzyme activity. Sampling coincided with recovery from a regional, multi-year severe drought, allowing us to document how the strength of climate legacies varied with contemporary conditions. We found increased investment in genes associated with resource cycling with historically higher precipitation across the gradient, particularly in traits related to resource transport and complex carbon degradation. This legacy effect was strongest in seasons with the lowest soil moisture, suggesting that contemporary conditions—particularly, resource stress under water limitation—influences the strength of legacy effects. In contrast, investment in stress tolerance did not vary with historical precipitation, likely due to frequent periodic drought throughout the gradient. Differences in the relative abundance of functional genes explained over half of variation in microbial functional capacity—potential enzyme activity—more so than historical precipitation or current moisture conditions. Together, these results suggest that long-term climate can alter the functional potential of soil microbial communities, leading to legacies in carbon cycling.

## Introduction

Soil microbes are key drivers of biogeochemical cycling, releasing an estimated 75 Pg of carbon (C) from soil annually through decomposition [1] as well as creating and stabilizing up to 3.7 Pg C in soil organic matter [2–5]. However, the sensitivity of soil microbes and their functioning to soil moisture is not well understood, making it difficult to project changes under novel precipitation regimes that are expected to occur with climate change [6]. Moreover, patterns in soil microbial functioning across environmental conditions can be difficult to generalize due to the complexity of these communities, their high functional redundancy [7], and variable phylogenetic conservation of traits [8, 9]. Microbial traits, quantified with functional genes, may provide more tractable microbial community characteristics that drive rates of ecosystem processes and can be incorporated into modeling efforts [10–15].

Long-term climate may shape the ecological strategies of soil microbes, and therefore their trait distributions. Theories such as the Y-A-S framework aim to link environmental gradients to microbial traits relevant to soil carbon cycling, including resource acquisition, stress tolerance, and growth yield [13]. If historical climate creates informational legacies in the form of microbial trait composition, legacies in microbial community functioning may persist under novel environmental conditions [16, 17]. For example, under the same current moisture conditions, microbes from regions with higher mean annual precipitation (MAP) exhibit higher microbial enzyme activity [18], accelerated decomposition rates [19], and increased soil C fluxes [20, 21] relative to those with drier climate histories. Precipitation history can also alter the sensitivity of ecosystem processes to contemporary conditions, such as recovery from drought [22]. These historical contingencies in microbial function may occur if long-term differences in precipitation (e.g. MAP) lead to local adaptation in the genetic composition of microbial populations and communities, such as increased abundance of genes that confer stress tolerance (e.g. osmolytes, peptidoglycan cell walls, dormancy/sporulation) [23, 24]. Indeed, a recent study [25] found functional legacies across two aridity gradients, where historically wetter sites were associated with higher abundance of genes associated with resource degradation, motility, and growth yield, whereas historically drier sites were enriched in stress-related genes.

Climate legacy effects are found widely, but inconsistently [6, 26], emphasizing the need to explore the drivers of these legacy effects and the factors that modulate them. If long-term climate shapes the ecological strategies of microbial communities, particularly those related to resource acquisition and stress tolerance, these patterns in microbial traits may exert persistent effects on the rate and sensitivity of soil C cycling processes—leading to legacy effects [13]. Alternatively, contemporary conditions may lead to rapid reassortment of taxa and/or functional genes, undermining any legacies in microbial structure and function [27, 28]. Shifts in microbial trait composition resulted in drought legacy effects in a recent modeling study [29], but more empirical work is needed to understand when and under which conditions historical precipitation constrains microbial trait distributions.

In this study, we investigated how precipitation history shapes the abundance and composition of microbial functional genes along a MAP gradient, as well as whether previously observed legacies persisted following relief from a prolonged, severe drought across the region. To do this, we focused on soil microbial communities across the Edwards Plateau in Texas, USA, which is a well-studied ecoregion where MAP ranges from 400–900 mm across ~400 km. In this region—across which soils, vegetation and temperature vary minimally—previous studies consistently find that MAP is the primary driver of soil respiration, extracellular enzyme activity, and C use efficiency [18, 20, 30]. Moreover, microbial investment in resource acquisition (total potential enzyme activity) differed in its sensitivity to soil moisture across the rainfall gradient [18]. Functional and compositional differences between sites with different precipitation history persisted when exposed to novel rainfall treatments, suggesting that local microbial communities resist turnover and thereby contribute to the strong climate legacy in ecosystem processes [31, 32]. Yet the genetic traits underlying these persistent climate legacies remain unknown. Lower MAP and its associated rainfall deficits may select for investment in stress-tolerance traits, at the expense of those related to resource acquisition [13, 23, 33], thereby driving legacies in ecosystem-level processes. However, evidence for this tradeoff has been inconsistent [34, 35], and it is not well understood to what extent historical vs. current environmental conditions affect the distribution of these traits.

Here, we characterized soils from across the MAP gradient with shotgun metagenomics to assess how climate legacies shape the abundance of functional genes related to microbial stress tolerance and resource acquisition, two traits important to rates in soil carbon cycling [13], have been shown to trade off [36], and whose genetic determinants are sensitive to drought [37]. We focused on a period in 2015–2016 when wetter conditions induced by an El Niño event followed a long-term drought (2011–2015). By sampling repeatedly over three seasons, we were able to capture a variety of contemporary soil moisture conditions and contrast how precipitation history (MAP) versus current soil moisture conditions drive microbial functional potential, as defined by functional gene relative abundance. Previous work in this study system found climate legacies for both microbial community structure, and respiration rates that lasted up to 4.5 years after exposure to new rainfall regimes [31]. We therefore expected microbial genetic traits to be primarily driven by precipitation history rather than contemporary soil moisture, with genes related to stress tolerance dominating historically drier sites and genes for resource acquisition more abundant in historically wetter sites. Local variation in microbial communities associated with long-term precipitation history could result in distinct community functional potentials that differentially constrain how sites respond to drought release, which would be indicated by an interaction between MAP and current moisture. Finally, we expected that functional genes would explain variation in microbial processes, including enzyme activity and soil respiration, indicating that differences in microbial functional potential are relevant for ecosystem-level fluxes.

## Materials and methods

### Study system

**Figure 1:**
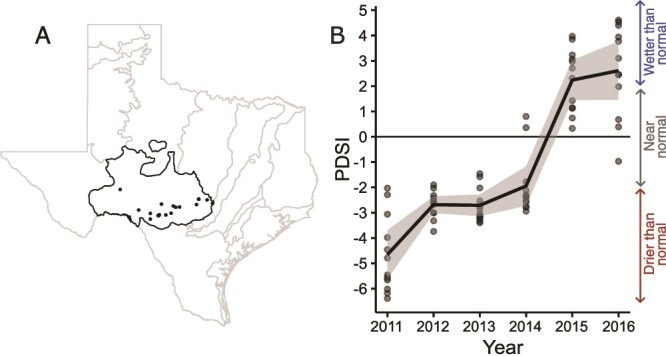
(A) Study sites (n = 20) were located across Edwards plateau (black outline) in Texas. (B) Palmer Drought Severity Index for each site based on monthly values across the Edwards Plateau. Soil sampling occurred in fall 2015, spring 2016, and summer 2016. Drought index data were retrieved from https://wrcc.dri.edu/wwdt/, accessed 24 September 2024 and are summarized in Supplementary data Sheet 2.

Soils were collected seasonally from 20 savanna grassland sites (Table S1) located across a steep precipitation gradient (400–900 mm MAP; 30-year norms 1981–2010 retrieved from PRISM, https://prism.oregonstate.edu/normals/) on the Edwards Plateau in central Texas, USA (Fig. 1A). As described elsewhere [20, 30], soils on the Edwards Plateau are derived from a single geologic formation and are all shallow, rocky, calcareous Mollisols. 30-year mean annual temperature varied from 17.7–20.6°C across the 20 sites. Previous studies across this gradient [20] did not find a correlation between soil properties and MAP, allowing us focus on long-term climate as a primary driver of differences in microbial traits and functioning across the gradient. The region experienced a severe multiyear drought between 2011 and October 2015 (i.e. Palmer Drought Severity Index −4.1 to −0.32011–2014), which was ended by an El Niño Southern Oscillation (ENSO) event in October 2015 through 2016 (PDSI 2.9 to 4.0) (Fig. 1B).

### Sample collection and processing

To capture the period of recovery from drought, soils were collected from the same locations at each of 20 sites at three dates (seasons): November–December 2015 (fall), March–April 2016 (spring), and June 2016 (summer). At each site, soil samples were collected from two 20 × 20 m plots with at least 50% grass cover and slope < 2% to minimize differences between sites. To collect sufficient soil to represent each large plot, subsamples were collected systematically from 100 points per plot by digging with a trowel to 15-cm depth systematically across the grid, then sieved to 1-cm in the field to remove rocks and homogenize. This resulted in a minimum of 4 L of soil, which was then subsampled into bags for DNA extraction, biogeochemical analysis, respiration assays, or enzyme assays, and stored on ice for transport to the lab.

### Shotgun metagenomics

Soils were stored at −80°C until total genomic DNA was extracted from 0.25 g from each plot using DNeasy Power Soil Kits (Qiagen, Germantown, MD, USA). DNA extracts were quantified fluorometrically (AccuClear Ultra High Sensitivity kit, Biotium, Fremont, CA, USA), and normalized to 0.5 ng/μl, and combined at the site level. Libraries were prepared as previously described [38] using Nextera adapters and sequenced (2 × 150 bp) on a NovaSeq 6000 System (Illumina, San Diego, CA, USA) at the NC State University Genome Sequencing Lab.

### Biogeochemical analysis

Soils were stored at 4°C for less than 24 h before sieving to 2 mm, and estimating microbial biomass C via chloroform fumigation and extraction in 0.5 M K_2_SO_4_ [39], as well as pH measurement in a ratio of 2:1 soil to water (Van Lierop 1990). Air-dried soils were used for texture determination via the hydrometer method [40]. The remaining sieved soil was oven-dried at 105°C for at least 48 h (to constant weight) for measurement of gravimetric soil moisture and analysis of soil total organic C and total N via combustion on a CHNS Analyzer (Perkin Elmer 2400, Waltham, MA, USA). All biogeochemical measurements were carried out at the plot level and averaged for site-level analysis (Supplementary data, Sheet 3).

### Microbial respiration

Soils for respiration assays were air dried to 5% gravimetric moisture. To measure CO_2_ flux under water-replete conditions, we constructed four replicate microcosms containing 25 g of soil from each sample. Soils were selected from one plot per site, chosen by coin flip. The microcosms were 60-ml borosilicate glass tubes (I-Chem, ThermoFisher Scientific, Waltham, MA, USA) with a septa cap. Soils were adjusted to 18–20% soil moisture with sterile water and maintained by weight for 8 weeks. To assess respiration, the microcosm headspace was flushed with CO_2_-free air and sealed for 1 h prior to sampling. CO_2_ was quantified on a gas chromatograph equipped with a methanizer and FID detector (SRI Instruments, Torrance, CA, USA). CO_2_ was measured biweekly and averaged across time and replicates for site-level analysis (see Supplementary data, Sheet 4).

### Extracellular enzyme activities

Soils for enzyme assays were stored at −20°C. Hydrolytic enzyme potential activities were measured for α-glucosidase, β-glucosidase, β-xylosidase, cellobiohydrolase, N-acetyl glucosaminidase, and acid phosphatase using fluorometric substrates [41, 42]. We focused on hydrolytic enzymes because we expected that the hydrolysis process would be affected by both short- and long-term rainfall, which is supported by our previous work [18]. Briefly, 2 g soil were blended with acetate buffer to create a slurry, from which eight technical replicates were then incubated with substrate for 1 h at 26.5°C. Fluorescence was measured on a plate reader (Spectromax M3, Molecular Devices, San Jose, CA, USA) with excitation at 365 nm and emission at 450 nm. For this study, total enzyme activity was calculated as the log-transformed sum of all enzyme activities, which has previously been used as a measure of microbial investment in resource acquisition [18]. Enzyme measurements were made on soils from each plot and averaged for site-level analysis (Supplementary data, Sheet 5).

### Bioinformatics

After removing three samples with a low read count, the sequencing depth of the remaining 57 samples ranged from 17.4 million reads to 82.6 million reads. Raw reads were checked for quality in *FASTQC* (Andrews 2010), and PhiX reads were removed using *bowtie2* [43]. We removed Nextera adapters and low-quality sequences in *fastp* using the default settings (phred quality > = Q15, unqualified bases limit = 40%), [44]. Coverage of these trimmed sequences was estimated in R using the nonpareil package [45]. Reads were assembled into contigs within each metagenomic sample using *SPAdes* [46] and evaluated with *MetaQuast* [47]; only contigs longer than 1000 bp were retained. Contigs were annotated using *Prokka* [48] with the—metagenome option, and proteins were then functionally annotated using *eggNOG-mapper v2* [49].

Functional regions of interest were identified by KO annotations from the KEGG and CAZy databases. EggNOG entries assigned to multiple KOs were split into separate entries, as each KO is associated with a specific protein coding sequence [50]. Our analyses focus on bacterial and archaeal genes, as fungal and other eukaryotic genes were too low in abundance to include in analyses. We categorized genes into functional groups of interest using the KEGG KO designations used by the *microTrait* tool in R, which was created to evaluate functional traits in microbial genomes [51]. Because the *microTrait* tool is designed to run on full genomes, we instead extracted the lists of KEGG genes associated with each trait (Supplementary data, Sheet 6). This identified genes associated with resource acquisition and stress tolerance, as well as with more specific functions within these broader categories using the level 1 *microTrait* designations [52]. Using level 1 maximized KOs with a trait designation, as many genes lacked a more granular categorization. The trait category “resource use”, referring to growth yield, was not used in our analyses due to few genes being assigned to that category.

### Data preparation

To prepare for our analyses, we first assessed soil properties as a function of MAP and season via linear models to ensure that there was sufficient independent variation in contemporary conditions to contrast with historical conditions. For these environmental characterizations, a Bonferroni-corrected alpha of 0.008 was used to account for multiple testing. Second, for analysis of functional genes, raw gene counts were converted to relative abundances by dividing by the total number of KEGG genes across each sample, and multiplying by 100. When examining different functional groups of genes, their relative abundances were summed.

### Statistics: analysis of historical vs. contemporary effects on functional genes

We assessed how precipitation history (MAP) influenced microbial functional genes, and how the strength of this effect varied through time and with contemporary conditions. Although we were interested in the persistence of this effect with increasing time since release from drought (i.e. sampling season), soil moisture varied strongly with sampling season. Therefore, we ran analyses with two full factorial configurations of explanatory variables to explore how these related, but distinct, drivers interacted with historical climate: MAP * Season, and MAP * Moisture.

We assessed the effect of these drivers on the composition of overall functional genes, stress tolerance genes, and resource acquisition genes using the *adonis2* and *metaMDS* functions in the *vegan* package [53]; when MAP was significant, distances from centroid were extracted with *betadisper* and regressed against MAP to confirm that dispersion was not driving trends. We ran simple linear regressions, using both the MAP * Season and MAP * Moisture structures, assessing how historical climate (MAP) and contemporary conditions (sampling season and soil moisture) affected the relative abundance of resource acquisition and stress tolerance genes. When MAP and soil moisture showed a significant interaction, we ran two follow-up models in which we converted one driver into a categorical variable [MAP: dry (< 600 mm/yr), mid (600–800 mm/yr), wet (> = 800 mm/yr); soil moisture: low (<= 10%), medium (10–20%), high (>20%)]. These follow-up models (MAP_Categorical * Moisture, and MAP * Moisture_Categorical) were used to aid interpretation of the intereaction of the two continuous variables. For these analyses, the *emmeans* and *joint_tests* functions in the *emmeans* R package were used for pairwise comparisons between sampling seasons [54]. We determined whether there was evidence of a tradeoff between the abundance of stress tolerance and resource acquisition genes, expecting a negative correlation if investment in these traits involve tradeoffs. For this analysis, we used a Pearson correlation within each sampling date in the *rstatix* R package [55].

To analyze specific gene functional categories within the two broader stress tolerance and resource acquisition functional groups, we ran multiple linear models using the *manylm* function in the *mvabund* R package [56], using the same full factorial model structures described above (MAP * Season and MAP * Moisture). We limited our focus to gene subcategories that were represented in at least 90% of samples, resulting in retention of 13 of the 21 resource-related and five of the seven stress-related functional subcategories—retaining 18 categories in total. When MAP and soil moisture significantly interacted to affect gene abundances, we visualized these relationships with the *interactions* R package [57]. For the above analyses, we set alpha at 0.05.

### Statistics: Linking gene abundances with function

If genetic differences underlie climate legacy effects, we expect the relative abundance of functional genes to explain variation in CO_2_ flux and enzyme activity across the rainfall gradient. Therefore, we assessed the effect of gene functional abundance on these fluxes and compared this effect to current (soil moisture) and historical (MAP) precipitation variables. To investigate linear relationships between environmental variables and functional gene categories, we used residual randomization in permutation procedures (RRPP), a method that performs non-parametric ANOVA on multivariate data by comparing fitted model coefficients to pseudo values generated from null model residuals across many permutations, using the *lm.rrpp* function in the *RRPP* R package [58]. We constructed models using the abundances of the most abundant 18 functional gene categories (see above), MAP, soil moisture, and sampling season as predictors of CO_2_ flux and total enzyme activity (with type III sums of squares and 10 000 iterations). To avoid overfitting the models, we did not include interaction terms; preliminary linear models suggested there was no significant interaction between MAP and either sampling season or soil moisture, for either response variable. We compared the *R*^2^ of models with genes only, environment only (MAP, season, soil moisture), and with both genes and environment combined, and used the *model.comparison* function to compare models based on AIC. The *tidyverse* [59] and *paletteer* R packages [60] were used for data manipulation and visualization.

## Results

### Variation in contemporary conditions

Soil moisture varied strongly across sampling dates (*P* < .001) peaking in Fall 2015 (average across sites: 23.6%) and declining at later dates (Spring 2016 = 14.7%; Summer 2016 = 12.5%; Fig. S1). Soil moisture did not vary significantly with MAP across these sampling dates (Fig. S2), allowing us to contrast the effects of historical rainfall (MAP) vs. contemporary conditions (soil moisture) on functional gene abundance (Table S2). When assessing other edaphic factors, microbial biomass C was higher in the Spring (0.532 mg/g) compared to both Fall 2015 (0.209 mg/g) and Summer 2016 (0.288 mg/g; *P* = .002). Soil pH decreased with MAP (*P* < .001) and measured values ranged from 6.7 to 8.4, contrasting with previous characterizations of this rainfall gradient [30]; however, the relative abundances of stress tolerance genes and resource acquisition genes did not vary significantly with pH (Table S3). Soil C, N, and texture were unrelated to either MAP or season (Table S2).

### Gene composition varied with MAP

**Figure 2:**
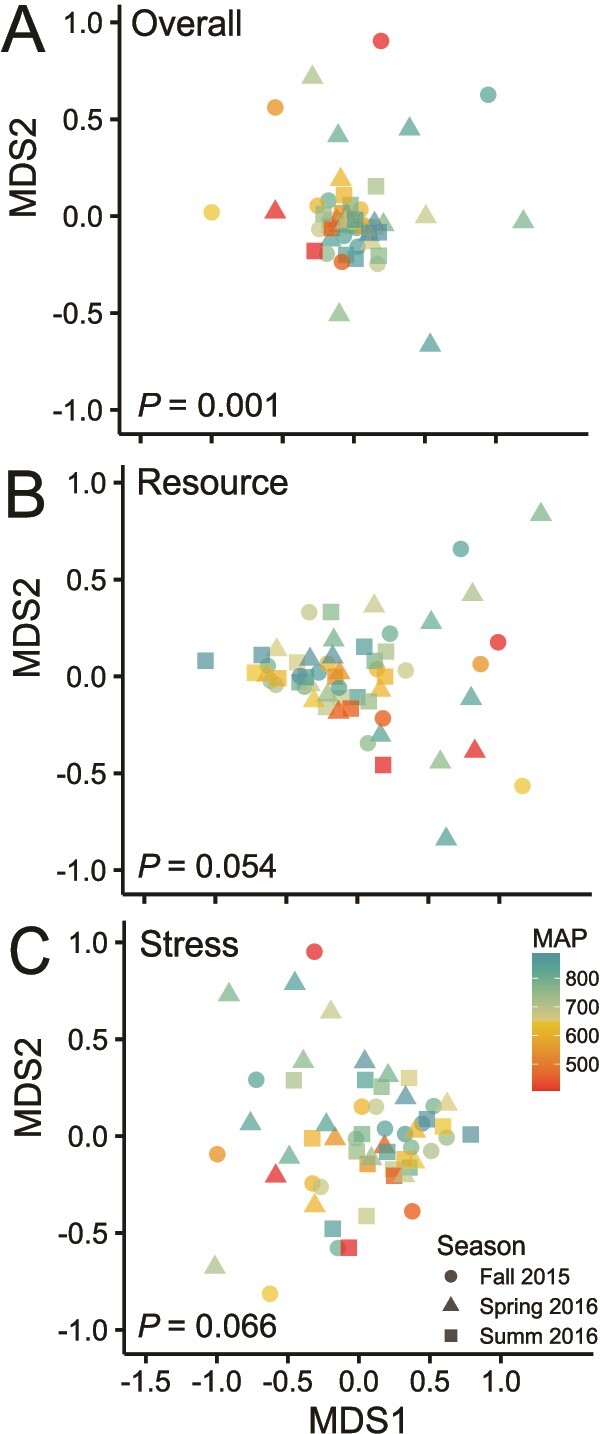
NMDS of (A) all KEGG genes, (B) resource acquisition genes, and (C) stress tolerance genes across the rainfall gradient. Based on PERMANOVA, the composition of functional genes significantly varied with MAP (color) for both (A) all KEGG genes, whereas the trend was similar but not significant for (B) resource acquisition genes and (C) stress tolerance genes. There were no differences in functional gene composition among seasons (shape).

Annotated contigs yielded 126 884 bacterial and 38 513 archaeal genes in the KEGG database with at least 10 occurrences, with 3028 unique KEGG KO designations. Overall gene composition varied minimally with MAP (PERMANOVA: *R*^2^ = 0.04, *P* = .001; Fig. 2A) and did not differ across seasons; when soil moisture was used as a predictor instead of sampling season, MAP was also a significant predictor of gene composition (Table S4). We identified 316 resource acquisition genes and 63 stress tolerance genes with a minimum of 10 occurrences (Supplementary data, Sheet 7). Trends were similar when rare genes were retained. The genetic composition of microbial communities was also shaped by MAP when aggregated into *microTrait* level 1 functional categories, although the effect was also limited (PERMANOVA: *R*^2^ = 0.05, *P* = .013; data not shown; Karaoz and Brodie 2022).

### Climate legacies in investment in resource acquisition

**Figure 3:**
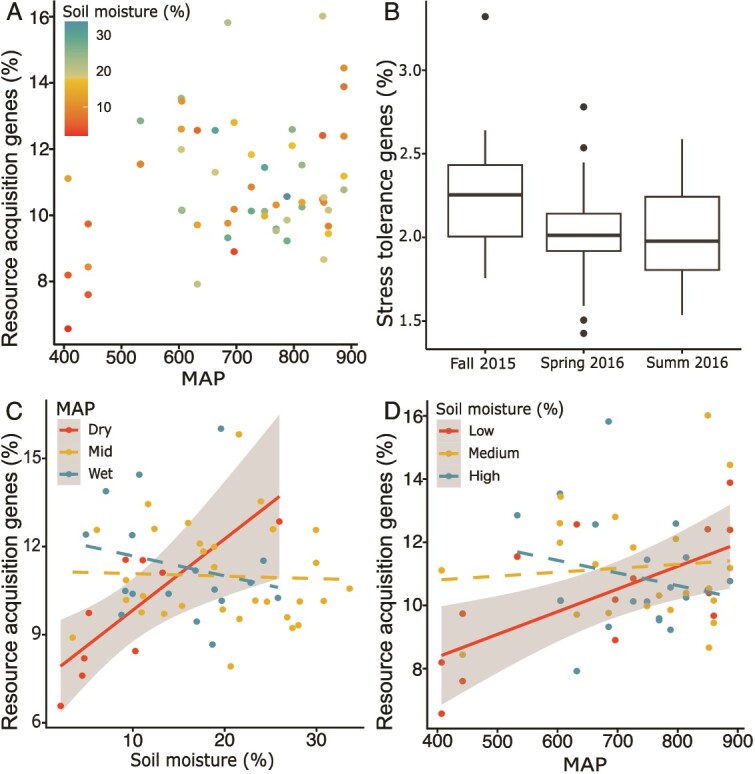
(A) Percent of genes associated with resource acquisition across the MAP gradient, with points colored by contemporary soil moisture. (B) Percent of genes associated with stress tolerance in each sampling season. (C–D) interaction between MAP and soil moisture in resource gene abundance: There was higher sensitivity to soil moisture in historically dry sites (400–600 mm/yr) than mid (600–800 mm/yr) or high-precipitation (>800 mm/yr) sites (C). Similarly resource genes increased with MAP under low contemporary soil moisture (< 10%), but not under medium (10–20%) or high (> 20%) soil moisture conditions (D).

Similar to overall gene composition, the composition of resource-acquisition genes tended to vary with MAP, albeit less strongly (PERMANOVA *R*^2^ = 0.03, *P* = .054, Fig. 2B), but not with sampling season or with soil moisture (Table S4). The total relative abundance of all genes related to resource acquisition was unaffected by MAP, sampling season or their interaction in the MAP * Season linear model. However, there was a significant interaction between MAP and soil moisture on the abundance of resource-acquisition genes in the MAP * Moisture model (*P* = .003, Fig. 3A, Table S5). To explore this interaction, we ran the same model, but with these predictors broken up into categories. When sites were split into three MAP categories, the relative abundance of resource acquisition genes increased with soil moisture at the driest sites (MAP <600, *P* = .007; Fig. 3C). Similarly, when soil moisture was split into three categories, resource genes increased with MAP under the driest conditions (soil moisture <10%, *P* = .007, Fig. 3D).

**Figure 4:**
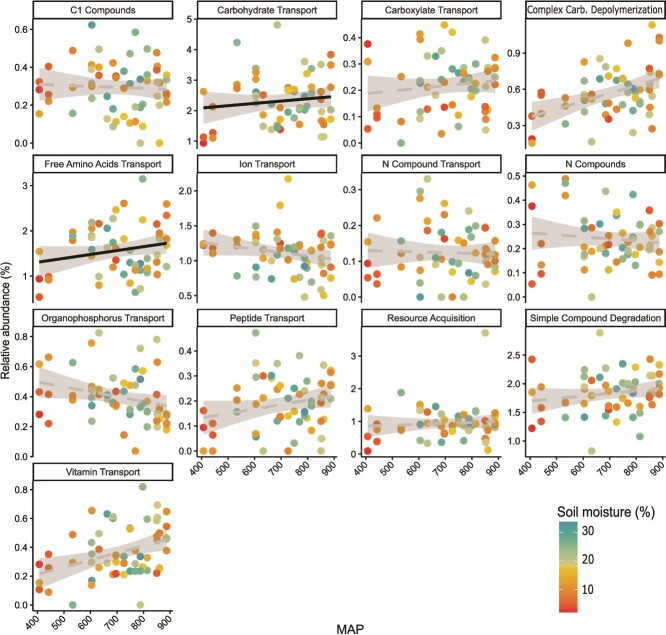
Relative abundance of resource genes associated with specific functions, as a function of MAP (x-axis) and soil moisture (point color). Of the 13 most abundant functional categories, free amino acid transport increased with MAP (*P* = .010, solid black trendline) and was influenced by an interaction between MAP * moisture (*P* = .032). A similar but marginally significant interaction was seen with carbohydrate transport (MAP: *P* = .085, dotted grey trendline; MAP * moisture *P* = .069).

MAP was a significant predictor of the relative abundances of genes in the resource acquisition subcategories in both model arrangements (seasonal model: *P* = .040, moisture model: *P* = .009) and interacted significantly with soil moisture (*P* = .045). When analyzed for patterns in univariate gene categories, genes associated with free amino acid transport and carbohydrate transport showed a tendency to vary with MAP, soil moisture, and their interaction, similar to the pattern shown with overall resource acquisition genes (MAP: *P* = .010 and *P* = .085; interaction: *P* = .032 and *P* = .069, respectively, Fig. 4, Table S6).

### Investment in stress tolerance varies with sampling season but not historical rainfall

The overall composition of stress tolerance genes varied slightly, but not significantly with MAP (*R*^2^ = 0.03; *P* = .066), and did not vary with either sampling season or soil moisture (Table S4). Relative abundance of stress tolerance genes did not vary with MAP but was highest in fall 2015 (*P* = .016; Fig. 3B); no driver was significant in the MAP * Moisture model (Table S5). No subcategory of genes related to different stress tolerance functions varied significantly with MAP and sampling season, or with MAP and soil moisture (Fig. S3, Table S7).

There was no significant correlation between stress tolerance and resource acquisition genes in any sampling season (all *P* > .2; Table S8; Fig. S4).

### Gene functional potential explains variation in functional assays

Both soil CO_2_ flux and total enzyme activity increased with MAP (*P* < .001 and *P* = .007, respectively); enzyme activity also varied with soil moisture (*P* > .001; Table S9). For enzyme activity, this “environment-only” RRPP model including MAP, sampling season, and soil moisture explained 43% of variation (Table 1; Table S9). However, the abundance matrix of the 18 functional gene categories explained more variation (55%) on its own. Including the gene matrix in the full model improved the *R*^2^ to 0.67, although an AIC comparison favored the more parsimonious environmental model.

Similarly, functional genes explained 47% of variance in CO_2_ flux, although the overall gene matrix term was only marginally significant (*P* = .064, Table S9). An environmental model (MAP + Season + Moisture) explained only 40% of the variation. Including the gene matrix in the environmental model increased the *R*^2^ to 0.63, although it was not favored by AIC model comparison, which heavily penalizes models with more parameters.

## Discussion

Microbial functional legacies related to precipitation history limit our ability to predict future C cycling using current moisture functions [6]. Extreme climate events, such as a regional multi-year drought, represent a possible tipping point whereby microbial communities undergo community re-assortment, overwhelming any legacies from long-term differences in climate [61]. However, in our study, long-term precipitation history (MAP) remained a primary driver of microbial functional traits (genes) despite regional relief from a long-term drought (Fig. 2). We found that climate history altered the sensitivity of resource acquisition gene abundance to current soil moisture, with the strongest effect of MAP under contemporary dry conditions (Fig. 3). Although investment in resource acquisition varied with both historical rainfall and current soil moisture, investment in stress tolerance did not respond to either of these drivers. The relative abundance of genes associated with these two traits did not correlate negatively at any sampling season, adding to evidence that Y-A-S strategies do not consistently trade off in microbial communities, at least at the genetic level [34, 35]. The composition of functional genes also explained much of the variation in microbial function, particularly total potential enzyme activity, suggesting that microbial genetic legacies underlie patterns in rates of ecosystem processes across the rainfall gradient.

**Table 1:** Comparison of model performance of total potential extracellular enzyme activity and soil CO_2_ flux rates, with different sets of predictors. Model compared include those using just gene abundances (Genes), abiotic drivers including MAP, soil moisture and season (Environment), and the full model containing both of these (Genes + Environment).

	Extracellular enzyme activity			Soil CO_2_ flux		
Model	*R* ^2^	*P*	AIC	*R* ^2^	*P*	AIC
Genes	0.55	**0.009**	99.320	0.47	*0.064*	−169.700
Environment	0.43	**<0.001**	84.519	0.40	**<0.001**	−191.425
Genes + Environment	0.68	**0.002**	89.352	0.63	**0.006**	−183.173

### Climate legacies shape investment in resource acquisition

Precipitation history (MAP) constrained overall microbial functional gene composition largely through the relative abundance of genes associated with resource acquisition. Previous work in this system documented a legacy of higher microbial extracellular enzyme activity and accelerated rates of soil respiration from soils from higher MAP sites, as well as altered sensitivity to current soil moisture conditions [18, 20]. Similarly, we found that the abundance of resource-associated functional genes increased with increasing MAP across the gradient, and that this effect was mediated by contemporary soil moisture (Fig. 3). Recent work in other aridity gradients also document more investment in resource acquisition traits in wetter sites, which they attribute to more available resources in historically wetter areas [25]. Our results add to evidence that precipitation history may frequently shape microbial traits via resource availability, while emphasizing that legacy effects may be hard to consistently detect due to fluctuating contemporary conditions.

The relative abundance of resource acquisition genes, as well as several resource transport gene sub-categories, increased with soil moisture at the driest sites (<600 mm precipitation/year) in our study, and were resistant to soil moisture changes at middle- and high-MAP sites (>600- mm precipitation/year, Fig. 3C-D, Fig. 4). This suggests that long-term precipitation history moderates the sensitivity of microbial functional potential to dry-down/wet-up events. Previous work has suggested that long-term exposure to drought conditions can alter microbial traits associated with C use, resulting in altered sensitivity of C fluxes [62]. In the case of our study, historical dry conditions may have favored populations with high uptake capacity, allowing microbes to quickly regulate osmotic balance, as well as take advantage of resource pulses associated with wet-up events [37, 63]. Indeed, other studies have documented increased potential growth rate of microbes exposed to a history of water stress [64], a strategy that would be facilitated by resource acquisition traits. Previous studies in our system demonstrated that, although the sensitivity of CO_2_ flux to soil moisture increased with MAP [20], extracellular enzyme activity was most sensitive at the drier end of the gradient [18]—a pattern similar to the resource acquisition gene response in our study (Fig. 3C-D). The patterns in our study, combined with these similar patterns in extracellular enzyme activity, point to resource limitation as a primary effect of soil moisture deficits, as well as a driver of microbial strategy. More generally, our results show that precipitation history can modify microbial genetic traits, and that these effect scale up to community-level functioning.

The interaction between contemporary moisture and MAP in resource acquisition gene abundance appears to be driven in part by transporter gene abundance, which also increased with soil moisture at historically drier sites with lower MAP (Fig. 4). In the Y-A-S model, the resource acquisition (A) strategy has been associated with increased resource uptake—which would favor increased transporter capacity—and/or resource degradation, which would favor extracellular enzyme production and depolymerization genes [13]. The responsiveness of transport functions in our study supports the first strategy. Other work [65] has suggested that a high abundance of membrane transporter genes can indicate increased microbial uptake of bioavailable C and a copiotrophic lifestyle. Microbes in xeric habitats, like those at the drier sites of our MAP gradient, may invest in transporters to facilitate rapid uptake of resources upon rewetting [24, 33]. Consistent with this explanation, previous work in permafrost cores saw similar rapid increases in functional genes for carbohydrate and amino acid transporters with freeze/thaw cycles [66], suggesting that disturbances can quickly alter microbial uptake capability. Short- and long-term variation in water availability may have distinct effects on microbial resource availability. Contemporary soil moisture controls resource diffusion, microbial release of osmolytes, and cell death, whereas climate shapes litter quantity and quality, soil organic matter, soil water-holding capacity, and other factors shaping the long-term abundance, availability, and mobility of resources [63]. Therefore, future studies that tease out the effects of contemporary soil moisture vs. long-term historical precipitation, and each of their effects on microbial resource availability, may help clarify the complex effects of how legacy effects in microbial functional potential are mediated by contemporary conditions.

### Stress tolerance investment varied little with climate history

We found that the abundance of genes associated with stress tolerance was resistant to differences in both MAP and contemporary soil moisture. These results were in contrast with what we might expect from theory—namely, more genes associated with stress tolerance with lower short-term (soil moisture) and/or long-term (MAP) water availability [29]—and what was found in other studies. For example, declining soil moisture during seasonal drought in grasslands led to short-term elevation of stress tolerance genes, including those associated with cell wall peptidoglycan biosynthesis, sporulation, and heat shock proteins [67]. Over longer (decadal) timescales, across a rainfall gradient, increasing aridity resulted in more genes associated with osmoprotection and sporulation [68, 69]. Similar to results from all stress genes, we did not find significant trends with soil moisture or MAP in any specific stress response category.

The observed lack of climate legacies in stress-related genes adds to findings that long-term precipitation patterns do not consistently alter functional traits as predicted by theory [34, 35], or can sometimes alter traits in the opposite direction (e.g. fewer stress response genes with increasing aridity in certain functions; [68–70]. In part, this variation may be due selection on microbial taxa in more constant vs. more fluctuating moisture environments. All sites in our study region experience frequent drought and high precipitation variability despite differences in total annual precipitation, so microbial communities may be similarly resistant to water stress across the gradient [30]. Previous work on the Texas rainfall gradient found that most microbial taxa were habitat generalists and highly persistent—even after reciprocal transplant across MAP regions—further supporting the idea that stress tolerance is pervasive in this system [32]. It is also possible that we found no change in stress-related genes such as osmolyte production because diffusion limitation in these dry, mineral soils limits the usefulness of osmoregulation as a stress response [24].

### Gene composition helps explain microbial functional capacity

Differences in the abundance of soil microbial functional genes can help explain variation in larger-scale ecosystem processes across gradients or in response to disturbances [11, 15, 71]. In our study, both extracellular enzyme activity and soil CO_2_ flux increased with MAP (Table S9), consistent with previous results from this region [20] and with other studies showing that carbon cycling processes increase with MAP along rainfall gradients [72, 73]. However, our results also suggest that the abundance of functional genes helped explain patterns in microbial functioning, explaining around half of variation in extracellular enzyme activity—more so than MAP, contemporary soil moisture, and seasonal effects combined (Table 1). There was a similar pattern of high variation explained by the gene-only model for soil CO_2_ flux, though the term was only marginally significant (Table 1). Previous work has established strong links between activities of extracellular enzymes and the abundance of their corresponding functional genes [74]. We extend this result to show that, at a broader scale, transport and C-degrading genes can help predict total extracellular enzyme activity as a measure of microbial investment in resource acquisition. We also found a similar but nonsignificant pattern in soil CO_2_ flux. Previous work has described difficulties in linking complex, emergent functions such as soil respiration to gene abundances [3, 18, 75]. Overall, our results indicate that more broad characterizations of microbial community functional potential can be useful predictors of overall microbial carbon cycling activity.

### Limitations and opportunities

One challenge in detecting patterns in specific functional categories is the difficulty in categorizing genes. Different osmoregulation genes, e.g. have previously been found to respond in opposite directions with MAP [68], perhaps because osmolytes are critical for drought tolerance but also have many functions in microbial cells. In this study, we used a database (*microTrait* KEGG tables) linking microbial genes to resource acquisition and stress tolerance functions based on Y-A-S trait categorizations [33, 51]. This approach allowed us to interpret genes in a functional context, identify community-level patterns in investment, and identify tradeoffs among microbial investment in broad strategies. Yet it limited our analysis to traits with well-characterized genetic determinants, making it difficult to assess patterns such as growth yield [13, 51] that control the fate of soil C. We also analyzed relative gene abundances of contigs at the community level because community-aggregated traits are especially relevant to ecosystem-level processes and do not require taxa-specific knowledge [11]. However, this contig-level analysis prevented identifying genome-level traits such as growth rate and C use efficiency, as well as within-organism tradeoffs between functional traits. MAG-based approaches with deeper sequencing, potentially combined with activity assays such as quantitative stable isotope probing, could link microbial pathways to specific taxa and link patterns in functioning to changes in the microbial community [15, 76]. For example, recent work [77] showed that freeze–thaw cycles change the functional capacity of forest soils via changes in microbial community composition. Finally, our study focused on bacterial and archaeal communities due to low fungal sequencing depth, but fungal functional genes are important drivers of soil C responses to precipitation [78]. Future work should incorporate fungi as they control decomposition, build soil C and likely have distinct responses to soil moisture variability.

## Conclusions

Climate can exert functional legacies on soil microbial communities, resulting in altered ecosystem-level processes [20, 79]. Our study suggests long-term precipitation patterns (MAP) alter the capacity of microbial communities to take up resources via changes in functional gene abundance. Stress response genes, in contrast, did not vary significantly across the MAP gradient. Microbial community investment in resource acquisition and stress tolerance genes therefore showed no evidence of a tradeoff in our study. Patterns in functional gene abundances explained a high percentage of variation in potential enzyme activity, suggesting that long-term precipitation can shape the genetic strategies of microbial communities in ways that influence the rates of microbial-driven soil processes. Our results show that microbial traits can clarify patterns in soil processes and their sensitivity to soil moisture, and these traits can help explain climate legacies in ecosystem processes.

## Supplementary Material

Broderick_etal_SUPPL_INFO

Broderick_etal_SUPPL_DATA

## Data Availability

Raw sequence data were deposited in the NCBI Short Read Archive under PRJNA1085749. Bioinformatics code was modified from: https://github.com/Gian77/metaGAAP. Gene annotation data, gene abundance data, and code for data analysis in R is at: https://github.com/brods21/Texas-Rainfall-Gradient-Metagenomic. All other data generated during this study are included in the published article and its supplementary files.
