# Supplementary material for: Long-term climate establishes functional legacies by altering microbial traits
Source: ISME J. 2025 Jan 13;19(1):wraf005. doi: 10.1093/ismejo/wraf005 (PMC11805608; doi:10.1093/ismejo/wraf005)
Supplement: Broderick_etal_SUPPL_INFO [file broderick_etal_suppl_info.pdf]

Long-term climate establishes functional legacies by altering microbial traits

Caitlin M. Broderick, Gian Maria Niccolò Benucci, Luciana Ruggiero Bachega. Gabriel D. Miller, Sarah E. Evans, Christine V. Hawkes

Supplemental Information

Item	Description	Page
Figure S1	Histogram of soil moisture across seasons	2
Figure S2	Soil moisture vs. MAP for each season	3
Figure S3	Stress gene abundances vs. MAP for specific gene functions	4
Figure S4	Stress tolerance vs. resource acquisition genes in each season	5
Table S1	Site location, historical climate, and soil properties	6
Table S2	Linear model results for soil properties as a function of MAP and season	7
Table S3	Linear model results for pH effects on stress and resource genes	8
Table S4	PERMANOVA results for analyses of gene abundances by MAP and season or by MAP and soil moisture, including test of dispersion	9
Table S5	Linear model results for stress and resource genes abundances by MAP and season or by MAP and soil moisture	10
Table S6	Results of multiple linear regression for subset of resource genes by MAP and season or by MAP and soil moisture	11
Table S7	Results of multiple linear regression for subset stress genes by MAP and season or by MAP and soil moisture	12
Table S8	Pearson's correlation of stress and resource genes at each season	13
Table S9	Results of RRPP models for soil respiration and enzyme activity as a function of gene abundances and environmental factors	14
Methods	Supplementary *microTrait* methods	15

Figure S1. Histogram of soil moisture at each sampling season.

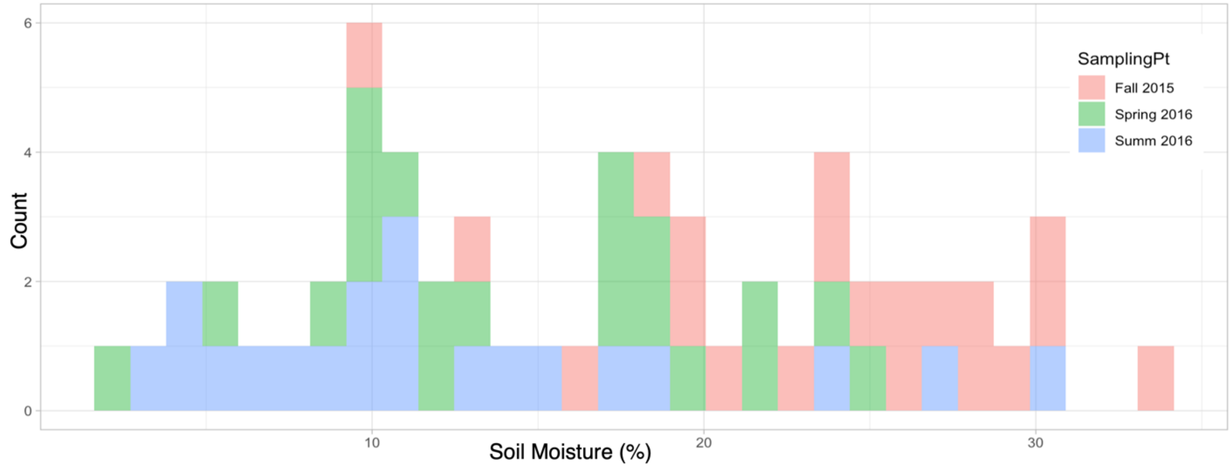

Figure S2. Soil moisture as a function of MAP at each sampling season. There was a marginally significant relationship with MAP across all seasons (P=0.064).

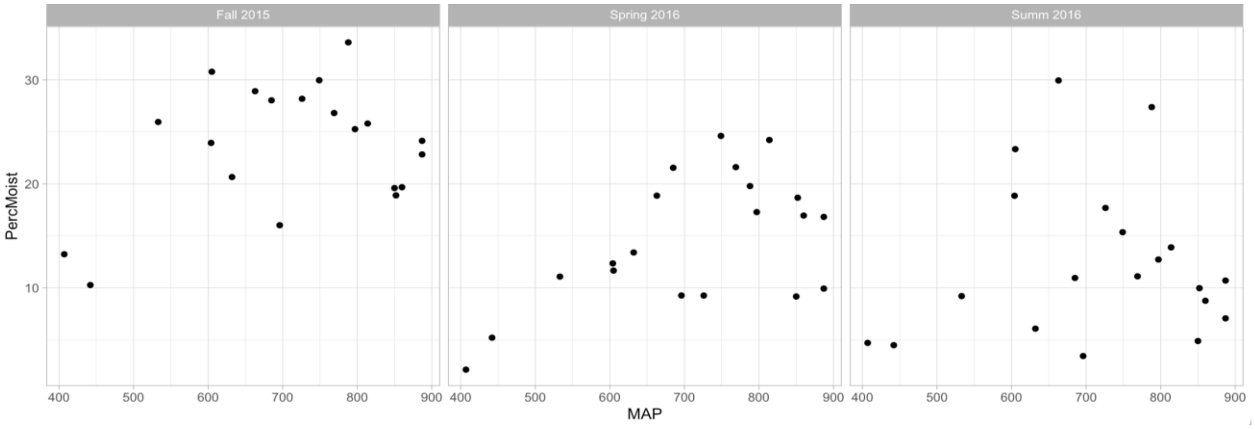

Figure S3. Relative abundances of stress genes associated with specific functions across MAP. There was no effect of either MAP or soil moisture on the investment of microbes in any specific functional category.

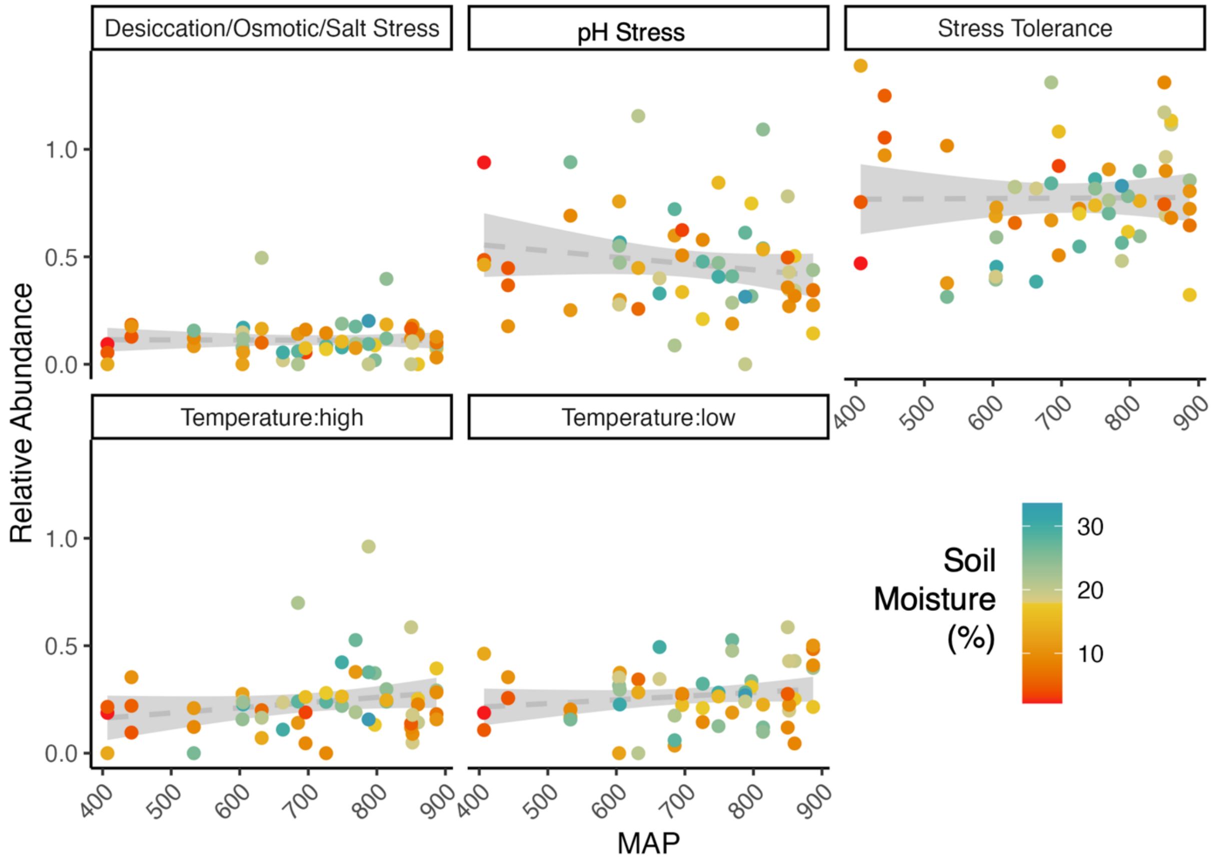

Figure S4. Relative abundances of stress tolerance genes vs. resource acquisition genes at each sampling season. There was not a significant relationship at any time point measured at any time point (Pearson P>0.05).

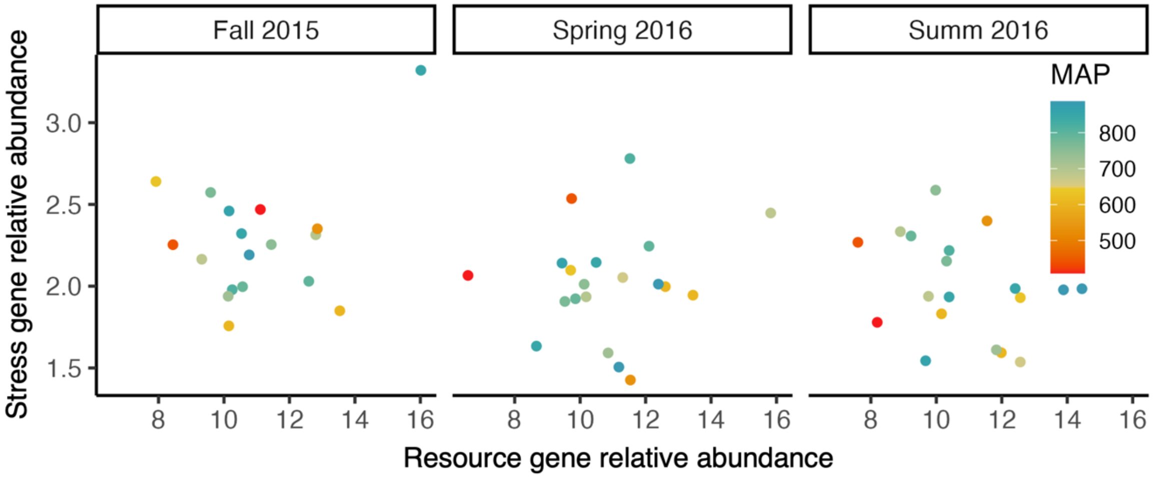

Table S1. Site location (Lat = latitude N, Lon= longitude W), historical climate based on 30-year means from PRISM (PRISM Climate Group, Oregon State University, https://prism.oregonstate.edu, data from 1981-2010, accessed 04/10/23). MAP is mean annual precipitation, MAT is mean annual temperature; basic soil properties are averaged over all three measurement dates.

Site	Lat	Lon	MAP (mm)	MAT (°C)	pH	C (%)	N (%)	Sand (%)	Clay (%)
FLASHS	30.67	-101.70	407	19.1	8.3	5.60	0.21	49.17	15.51
SCATSP	29.70	-101.32	442	20.6	8.0	3.59	0.13	25.19	26.48
DRITSP	29.94	-100.93	533	19.6	7.9	3.68	0.24	21.22	38.85
KCATSP	29.62	-100.45	604	19.7	8.0	4.68	0.27	25.10	37.50
SIEECO	29.82	-100.45	605	20.1	7.8	4.28	0.37	20.71	43.13
MORECO	29.74	-100.10	632	20.1	8.0	6.38	0.27	37.42	29.13
CASECO	30.15	-99.99	663	18.4	7.6	5.52	0.45	18.87	39.69
HENECO	29.75	-100.13	685	19.0	8.0	7.04	0.35	30.06	41.72
KOOECO	29.78	-100.08	696	18.9	8.3	10.41	0.18	47.34	20.59
KERWMA	30.08	-99.50	726	17.7	7.9	7.23	0.40	33.73	27.95
KENECO	30.04	-99.39	749	17.8	7.7	8.02	0.49	28.48	32.05
MOCECO	29.77	-99.81	769	18.5	7.9	3.81	0.27	16.84	33.48
SMIECO	30.05	-99.24	788	18.2	7.6	5.75	0.37	20.36	39.77
LMATSP	29.83	-99.59	797	18.2	7.8	5.26	0.44	21.12	40.96
COLECO	30.33	-98.44	814	19.1	7.6	5.82	0.33	44.47	22.35
BREECO	30.30	-98.10	850	19.3	8.0	10.29	0.33	47.20	15.30
INGECO	30.33	-98.45	852	19.1	7.3	1.25	0.11	57.83	15.00
KERECO	30.13	-98.53	860	18.4	8.0	11.37	0.20	37.86	18.76
CAWQPL	30.18	-97.87	886	19.9	7.1	2.79	0.24	24.61	25.79
LBJWFC	30.18	-97.87	886	19.9	7.0	3.15	0.26	21.08	37.67

Table S2. Linear model results for soil characteristics as a function of MAP, season, and their interaction. The smallest factor *P* value is reported for sampling season. Significant *P* values in bold indicate α < 0.008 (cutoff adjusted for multiple testing).

	**MAP**		**Sampling Season**		**MAP*Sampling Season**	
	t	*P*	*t*	*P*	*t*	*P*
Soil Moisture	1.334	0.188	-5.467	**<0.001**	0.724	0.472
Soil C	0.428	0.670	-0.248	0.805	0.341	0.735
Soil N	0.416	0.679	-0.475	0.636	0.414	0.681
Percent Sand	0.418	0.678	0.391	0.697	-0.441	0.661
MBC	-0.106	0.916	5.543	**<0.001**	1.121	0.267
pH	-3.084	**0.003**	1.667	0.101	1.304	0.763

Table S3. Statistical results for linear models between pH and the relative abundance of stress and resource genes. There was no significant trend for either category of gene abundances. Italicized *P* values are non-significant trends (0.05 < P < 0.1).

	**Stress Tolerance**		**Resource Acquisition**	
	t	*P*	*t*	*P*
pH	0.323	0.748	-1.927	*0.059*

Table S4. PERMANOVA results for analyses of gene composition (based on their relative abundances) by MAP and season or by MAP and soil moisture. Bolded *P* values are less than α = 0.05. Italicized *P* values are non-significant trends (0.05 < *P* < 0.1). Additionally, we extracted MAP distances from centroid for each MAP group using the betadisper() function, and then used a linear model to assess trends in dispersion across the MAP gradient. This step confirmed that dispersion did not vary as a function of MAP and that significant PERMANOVA results for MAP are robust.

**PERMANOVA results**												
	**Stress genes**			**Resource genes**			**All functional genes**			**Functional categories**		
	R^2	*F*	*P*	R^2	*F*	*P*	R^2	*F*	*P*	R^2	*F*	*P*
MAP	0.028	1.565	*0.059*	0.030	1.716	*0.054*	0.037	2.124	**0.002**	0.049	2.802	**0.013**
Season	0.039	0.871	0.271	0.037	1.079	0.294	0.038	1.013	0.181	0.017	0.488	0.944
MAP*Season	0.040	0.709	0.254	0.048	1.371	*0.086*	0.041	1.120	*0.096*	0.377	1.074	0.349
MAP	0.028	1.129	*0.066*	0.030	1.683	*0.059*	0.037	2.100	**0.002**	0.049	3.039	**0.008**
Moisture	0.016	1.048	0.577	0.013	0.735	0.767	0.015	0.865	0.730	0.026	1.602	0.108
MAP*Moisture	0.013	1.525	0.803	0.019	1.080	0.300	0.019	1.112	0.226	0.066	4.097	**0.001**
**Dispersion of MAP group distances**												
lm(dispersion~ MAP)	**Stress genes**			**Resource genes**			**All functional genes**			**Functional categories**		
	R^2	*t*	*P*	R^2	*t*	*P*	R^2	*t*	*P*	R^2	*t*	*P*
	-0.034	3.863	0.532	-0.055	0.261	0.797	-0.051	-0349	0.731	0.052	-1.413	0.176

Table S5. Results from linear models assessing relative abundance of total stress and resource genes as a function of (A) MAP*Season and (B) the MAP*Moisture. The lowest *P* value is reported for categorical descriptors and their interactions. Bolded *P* values are less α = 0.05. Italicized *P* values are non-significant trends (0.05 < *P* < 0.1).

(A)

	**Stress genes** (R^2=0.048)			**Resource genes** (R^2=0.000)		
	Estimate	*t*	*P*	Estimate	*t*	*P*
MAP	0.082	0.546	0.578	0.612	0.738	0.464
Season	-0.282	-2.487	**0.016**	-0.180	-0.292	0.772
MAP*Season	-0.141	-0.684	0.497	0.964	0.838	0.406

(B)

	**Stress genes** (R^2=0.002)			**Resource genes** (R^2=0.169)		
	Estimate	*t*	*P*	Estimate	*t*	*P*
MAP	0.080	0.771	0.444	0.016	0.032	**0**.975
Moisture	0.098	0.945	0.348	-0.135	-0.266	0.792
MAP*Moisture	0.348	1.693	*0.096*	-3.131	-3.012	**0.003**

Table S6. Results from multiple linear regression for the 13 *microTrait* categories of resource genes present in at least 90% of samples. These genes were analyzed as a function of both MAP*season and MAP*moisture. Bolded *P* values are less α = 0.05. Italicized *P* values are non-significant trends (0.05 < P < 0.1). Hooper's R^2 for the MAP * Season model was 0.116, and was 0.098 for the MAP * Moisture model.

	**Resource Acquisition (general)**		**C1 Compounds**		**N Compounds**		**Complex Carbohydrate Depolymerization**		**Simple Compound Degradation**		**N Compound Transport**		**Carbohydrate Transport**	
	F	*P*	*F*	*P*	*F*	*P*	*F*	*P*	*F*	*P*	*F*	*P*	*F*	*P*
MAP	0.008	0.966	0.803	0.919	1.355	0.832	5.094	0.256	0.629	0.921	2.027	0.747	0.260	0.966
Season	1.107	0.960	1.091	0.946	0.178	0.998	1.414	0.933	1.597	0.919	0.281	0.998	0.501	0.976
MAP*Season	0.133	0.980	1.564	0.886	2.584	0.741	0.100	0.996	0.930	0.940	1.940	0.834	2.268	0.785
MAP	1.235	0.892	0.043	0.991	0.090	0.910	5.773	0.187	0.674	0.954	1.699	0.839	7.773	*0.085*
Moisture	0.915	0.965	0.007	0.998	0.041	0.989	0.465	0.989	0.009	0.998	1.008	0.965	0.165	0.997
MAP*Moisture	1.343	0.909	0.000	0.993	0.892	0.943	0.382	0.986	0.050	0.993	2.582	0.702	8.098	*0.069*

(continued)	**Carboxylate Transport**		**Free Amino Acid Transport**		**Ion Transport**		**Organophosphate Transport**		**Peptide Transport**		**Vitamin Transport**		**Overall model**	
	F	*P*	*F*	*P*	*F*	*P*	*F*	*P*	*F*	*P*	*F*	*P*	*F*	*P*
MAP	1.696	0.786	0.194	0.966	0.068	0.966	4.588	0.306	3.467	0.458	7.545	0.103	27.73	**0.040**
Season	5.406	0.251	0.066	0.998	1.267	0.940	2.222	1.819	1.095	0.963	0.030	0.998	12.52	0.487
MAP*Season	4.390	0.389	0.546	0.945	2.399	0.755	1.060	0.940	1.119	0.940	1.460	0.886	14.39	0.373
MAP	0.031	0.992	12.885	**0.010**	0.219	0.991	0.143	0.992	3.752	0.433	1.320	0.892	35.637	**0.009**
Moisture	1.546	0.923	1.093	0.965	3.070	0.633	1.129	0.965	0.036	0.998	0.070	0.998	9.915	0.670
MAP*Moisture	0.388	0.986	9.861	**0.032**	0.054	0.993	1.021	0.942	1.725	0.860	0.169	0.988	26.566	**0.045**

Table S7. Results from multiple linear regression for the five *microTrait* categories of stress genes present in at least 90% of samples. Results for both the MAP*Season and the MAP*Moisture models are presented. For sampling season (categorical variable), the lowest *P* value for a level is presented. Adjusted *P* values were returned from the summary.manylm() using the Westfall and Young (1993) resampling algorithm and 10000 iterations. Bolded *P* values are less than α = 0.05. Hooper's R^2 for the MAP * Season model was 0.058, and was 0.066 for the MAP * Moisture model.

	**Stress tolerance (general)**		**Desiccation/ Osmotic/salt stress**		**pH stress**		**Temperature: high**		**Temperature: low**		**Overall model**	
	F	*P*	*F*	*P*	*F*	*P*	*F*	*P*	*F*	*P*	*F*	*P*
MAP	0.225	0.839	0478	0.839	0.372	0.839	2.864	0.364	1.218	0.681	5.157	0.379
Season	1.577	0.667	0.552	0.808	1.118	0.731	1.090	0.731	2.982	0353	5.305	0.378
MAP*Season	0.350	0.906	0.911	0.845	0.042	0.969	0.934	0.845	0.284	0.969	2.230	0.786
MAP	1.706	0.555	0.122	0.732	1.602	0.555	3.754	0.247	2.369	0.555	8.552	0.155
Moisture	1.352	0.661	0.205	0.832	0.750	0.750	4.284	0.190	0.298	0.832	6.889	0.248
MAP*Moisture	3.008	0.307	0.100	0.978	0.000	0.998	3.297	0.307	0.119	0.978	6.524	0.273

Table S8. Pearson's correlation (*r*) of stress and resource genes at each sampling season. There was no significant relationship at any season, suggesting a lack of a tradeoff between investment in these gene functions.

**Season**	***r***	**Statistic**	***P***
Fall 15	0.30	1.270	0.224
Spring 16	0.18	0.771	0.451
Summer 16	-0.29	-1.270	0.221

Table S9. RRPP PERMANOVA results explaining variation in total enzymes activity and soil CO_2 flux. Three models are considered: one with just the gene matrix, one with environmental variables (MAP, soil moisture, sampling season), and one with both sets of variables included. See Table 1 for overall model R^2, *P*, and AIC. Bolded *P* values are less than α = 0.05. Italicized *P* values are non-significant trends (0.05 < *P* < 0.1).

	**Enzymes**					**CO_2**				
	DF	SS	R^2	*F*	*P*	DF	SS	R^2	*F*	*P*
Genes model										
Genes	18	11.714	0.553	2.474	**0.009**	18	0.074	0.466	1.843	*0.064*
Environment model										
MAP	1	1.859	0.088	7.723	**0.007**	1	0.032	0.205	17.900	**<0.001**
Season	2	0.807	0.038	1.675	0.197	2	0.037	0.236	10.306	**<0.001**
Moisture	1	4.177	0.197	17.348	<**0.001**	1	0.007	0.045	3.933	*0.052*
Genes + Environment model										
Genes	18	5.207	0.246	1.355	0.225	18	0.026	0.162	2.425	**0.016**
MAP	1	0.523	0.024	2.452	0.125	1	0.024	0.101	3.381	**0.006**
Season	2	1.03	0.004	0.230	0.802	2	0.006	0.060	1.421	*0.076*
Moisture	1	0.840	0.040	3.935	*0.055*	35	0.019	0.374		

Supplementary methods

We assigned genes to *microTrait* traits using the methods described in Jones et al. 2024. Briefly, we used the KEGG KO designation from eggnog_mapper v2 output to match genes to the *microTrait* category designations. Because each eggnog hit sometimes had several functional domain/KOs, we split these entries so each KO was its own row. Microtrait provides a range of supplementry tables that link various genes and rules for assigning traits (Karaoz and Brodie 2022); these are referenced here. We combined the *microTrait* supplementary tables 5 and 6 by substrate, and removed substrates without a trait designation. We manually edited *microTrait* supplementary table 2 to add KEGG KO assignments to genes assigned by eggnog to a GH group from the CAZy database (Drula et al. 2021). We also treated osmolytes to the stress tolerance trait, as predicted by the YAS framework (Malik et al. 2020). Finally, we merged this modified supplementary table 2 with the previously-combined information from supplementary tables 5 and 5 by the *microTrait* HMM name. This final table (see Rules_microtrait sheet in Supplementary Data) was used to assign KOs to YAS categories. For analyses, we focus on the microTrait level 1 functional categories (i.e. microtrait_trait.name).
